# Time-Sensitive Network (TSN) Experiment in Sensor-Based Integrated Environment for Autonomous Driving

**DOI:** 10.3390/s19051111

**Published:** 2019-03-05

**Authors:** Juho Lee, Sungkwon Park

**Affiliations:** Both are with Department of Electronic and Computer Engineering, Hanyang University, Seoul 04763, Korea; ljh0122@hanyang.ac.kr

**Keywords:** autonomous driving, vehicle sensors, time-sensitive network, in-vehicle network

## Abstract

Recently, large amounts of data traffic from various sensors and image and navigation systems within vehicles are generated for autonomous driving. Broadband communication networks within vehicles have become necessary. New autonomous Ethernet networks are being considered as alternatives. The Ethernet-based in-vehicle network has been standardized in the IEEE 802.1 time-sensitive network (TSN) group since 2006. The Ethernet TSN will be revised and integrated into a subsequent version of IEEE 802.1Q-2018 published in 2018 when various new TSN-related standards are being newly revised and published. A TSN integrated environment simulator is developed in this paper to implement the main functions of the TSN standards that are being developed. This effort would minimize the performance gaps that can occur when the functions of these standards operate in an integrated environment. As part of this purpose, we analyzed the simulator to verify that the traffic for autonomous driving satisfies the TSN transmission requirements in the in-vehicle network (IVN) and the preemption (which is one of the main TSN functions) and reduces the overall End-to-End delay. An optimal guard band size for the preemption was also found for autonomous vehicles in our work. Finally, an IVN model for autonomous vehicles was designed and the performance test was conducted by configuring the traffic to be used for various sensors and electronic control units (ECUs).

## 1. Introduction

As autonomous vehicles are emerging, technologies including next-generation vehicle communication, vehicle to everything (V2X), and the advanced driving assistant system (ADAS), are also advancing. An autonomous vehicle communicates with traffic infrastructures, such as a nearby vehicle or RSU (roadside unit). The long-term evolution (LTE) and 5G communication technologies can be used as external communication technologies for autonomous vehicles. Dedicated short-range communication (DSRC) protocols are also used as external communication means for providing intelligent transport system (ITS) services. But, in order to exchange data between various sensors, processors, and ECUs (electronic control unit) in real time within a vehicle, in-vehicle networks (IVNs) also need proper broadband communications. Traditional IVNs include controller area network (CAN), local interconnect network (LIN), media-oriented systems transport (MOST), and FlexRay [1,2,3,4]. Recently, as traffic for autonomous driving increases, the application of Ethernet communication technology to vehicles is underway. This standardization work for autonomous vehicles has been going on since 2006 in the IEEE 802.1 TSN (time-sensitive network) [5]. The Ethernet TSN is still in the process of being developed. IEEE 802.1Q-2018 was completed in July 2018 [6]. A new version of 802.1Q will follow, and standard technologies for autonomous driving will continue to be reflected on.

IEEE 802.1Qbv and 802.1Qbu were published in 2015 and 2016, respectively. However, new contributions have been steadily emerging due to functional interoperability with linked standards and physical link scalability (IEEE P802.1ch Multi-Gig Automotive Ethernet PHY Task Force) when integrated into the 802.1Q standard [7,8,9]. The functions of these standards should be considered in the integrated environment to reconsider the parameters and requirements for the TSN environment in IEEE 802.1Q. Several studies have been conducted on performance testing on the IEEE 802.1 TSN through simulations and theoretical studies [10,11,12]. These studies have been conducted in an independent environment, which generally constitutes basic partial traffic in the TSN standards. Each of the standards being developed is independently published, and only the interworking of some of the most highly relevant standards are considered. Therefore, when integrating different standards into a single standard such as IEEE 802.1Q, there may be a lack of interworking and scalability between the main TSN functions in the standards. In some cases, some parameter values in the TSN standards may need to be changed.

Our contribution in this paper is the development of a TSN integrated environment simulator applying the main functions of the TSN standards such as IEEE 802.1AS, Qbv, Qcc, Qca, Qbu, and 802.3br that are in the early stage of standardization. Considering the upcoming revision of 802.1Q, in order to minimize the performance gaps that can occur when the functions of these standards operate in an integrated environment, we analyzed whether the traffic for autonomous driving satisfies the TSN transmission requirements in IVNs and whether the preemption function reduces the overall E2E (End-to-End) delay or not. Moreover, since there are so many different standards involved, as explained above, it is hard to predict whether a combined architecture with so many different parameters and configurations satisfy the requirements underlying autonomous vehicles. Software simulations enabled us to test many different possible scenarios with ease. This significantly saved us time and cost. In addition, as part of our analysis process in simulations, we derived a parameter for the preemption to work optimally. Finally, an IVN model to be used for autonomous vehicles was designed, and the performance test was conducted by configuring the traffic to be used in various sensors and ECUs. Actual data were provided by a major motor company in Korea.

The rest of the paper is structured as follows. Section 2 contains the overall trends in the development of TSN standards and a description of the two standards that will be one of the main focuses of this paper. Section 3 describes the TSN simulator in the developed integrated TSN environment and the main functions that can be changed through parameters. Section 4 deals with the traffic expected to be used in autonomous vehicles, and the simulation tests that were performed to check if the traffic would satisfy the TSN transmission requirements. Lastly, we conclude the paper and talk about future work in Section 5.

## 2. Background

The IEEE 802.1 TSN Task Group (TG) is part of the IEEE 802.1 Working Group (WG). The TSN TG evolved from the former IEEE 802.1 Audio Video Bridging (AVB) TG. The original work on AVB was completed in 2005. TSN standards have been newly published or revised based on the AVB standards since 2006, and the development status of major standards are shown in Table 1.

There are two representative standards that redefine the queuing process for time-sensitive traffic transmission. The first is IEEE 802.1Qbv-2015. The IEEE 802.1Qbv standard has extended the time-based scheduler for time-sensitive traffic transmission and its queuing procedure over AVB traffic transmission structure [7]. The transmission method of existing AVB streams was an event trigger. However, in a TSN, a time-aware scheduler is applied in addition to the existing method to transmit scheduled traffic (ST) by a time-trigger transmission method. ST is transmitted at regular intervals in the vehicle as important data related to vehicle control and safety. If the transmission requirement of the existing AVB stream is to deliver within the maximum 2 ms time delay when passing seven hops, the transmission requirement of the ST is to deliver within 100 μs time when passing five hops. A hop represents a switch. All nodes (switches, gateways, sensors, or ECUs) must be synchronized to the same clock with almost no error in order to transmit the ST properly based on the transmission time. Therefore, a time-aware scheduler should be applied with the time synchronization function of the IEEE 802.1AS-rev standard [13]. In this paper, we refer to this standard and implement these functions in the simulator.

**Figure 1:**
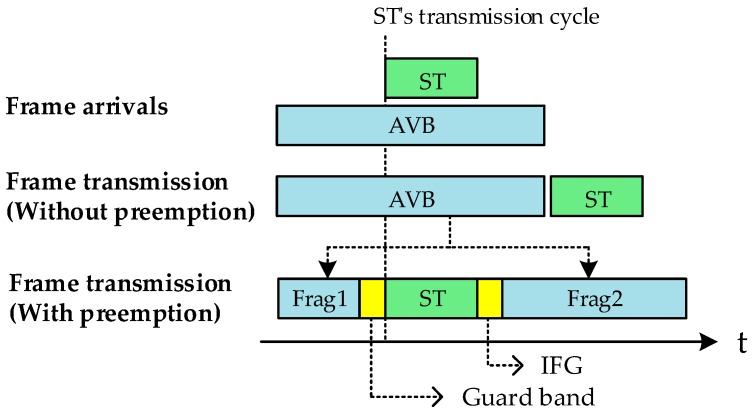
Example of frame transmission with preemption.

The second standard is IEEE 802.1Qbu-2016 [7]. IEEE 802.1Qbu is the standard that defines the preemption mechanism. In the existing transmission policy, if there is a frame being transmitted, the frame is designed to wait until the ongoing transmission is finished, even if there is a frame that needs to be transmitted urgently. However, with the preemption technique, high-priority frames can be preferentially processed. The preemption may interrupt the transmission of a low-priority frame (AVB) during transmission to preferentially process a time-sensitive frame (ST). After processing the high-priority frame, it resumes transmission of the low-priority frame and receives it in the full frame. When switching gates for transmission between ST and AVB, a “guard band” is set for a certain time window to prevent frame collision (see Figure 1). Low-priority frames can be divided into two or more frames as needed.

In order to transmit the ST at the reserved time, there is another method of blocking the frame (1500 bytes of maximum frame size) from entering before the transmission cycle of the ST. However, it is inefficient in utilizing network resources and also increases the delay time of non-ST frames. Therefore, the preemption is required to increase network efficiency and reduce overall delay time. The standard technologies mentioned above have been consistently conducted with the standardization work. In general, theoretical analysis of the time-aware scheduler of a TSN and experiments measuring IVN performance through simulation are the main subjects. Recently, there have been studies analyzing the performance of the preemption [14,15,16,17,18].

## 3. TSN Simulator Development

In order to develop the integrated IEEE 802.1Q standard simulation, we implemented the main TSN functions in the published standards such as IEEE 802.1AS, Qbv, Qcc, Qca, Qbu, and 802.3br. Section 3 introduces the time-aware scheduler functions of IEEE 802.1Qbv-2015 (which are the main TSN functions of a TSN) and the implementation issues of the frame preemption of IEEE 802.1Qbu-2016 and 802.3br and describes the configuration of each variable operation parameter related to it. The simulator has been developed based on OMNET ++ version 5.0 and inet-3.3.0, and some related studies that have been conducted can be found in References [10,11,14,19].

### 3.1. Time-Aware Scheduler

**Figure 2:**
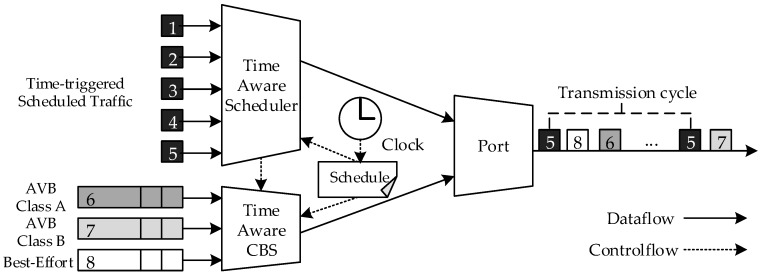
Time-aware scheduler and queuing structure in the in-vehicle network (IVN) nodes.

The time-aware scheduler acts as a timetable for transmitting data at fixed intervals without being influenced by the transmission of AVB or BE (best effort) traffic. To achieve this, it is important to set a cycle for each stream and appropriately place the ST within the cycle. For this purpose, a parameter for specifying a period for each stream and a parameter for allocating a transmission start time of each ST within the period were separately configured. Since this series of operations was based on the assumption that all nodes in the vehicle are synchronized, a periodic synchronization was performed by designating a specific node as a master, which follows the IEEE 802.1AS-rev operation. Each node was designed to have eight queues—five for ST, two for AVB (Class A, B) traffic, and one for BE traffic (see Figure 2).

### 3.2. Frame Preemption

The principles of the frame preemption and development issues in implementation are as follows. In order to start the transmission through the time-aware scheduler, the ST stops transmission of the frame that was being transmitted and splits the frame up at the transmission stop point to transmit the ST first. When the transmission of the ST is completed, the frame preemption functions to transmit the frame that the ongoing transmission interrupted. When the transmission of the ST is completed, the frame preemption successively transmits the interrupted frame and restores the original frame. The key point of the frame preemption is to stop the non-ST frame in transit and to combine the whole of the divided frame. The guard band is the interval between the non-ST frame to be interrupted and the ST frame to be transmitted (see Figure 1). The guard band is processed in units of data size.

**Figure 3:**
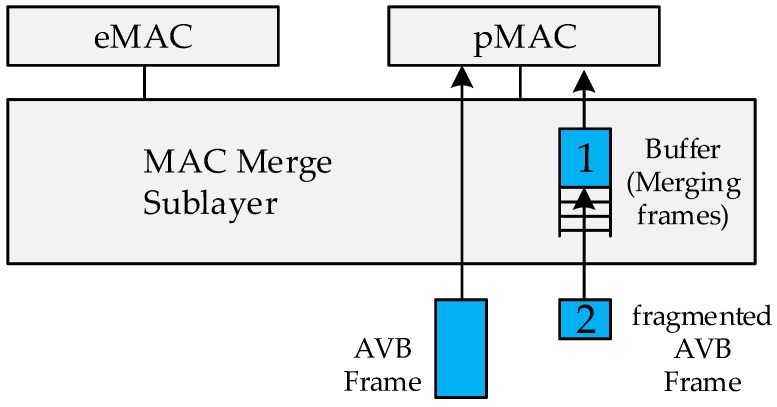
Receiver side MAC merge sublayer structure.

In order to apply the frame preemption, frame division and merging processes should be implemented by referring to IEEE 802.3br standards [20]. The preemption process of dividing and merging the frames is performed in Layer 2 because the PHY (Physical) layer cannot recognize the operation of the preemption. Therefore, logical layer processing is required in the MAC (Media Access Control) merge sublayer when going up to Layer 2 (see Figure 3). In Layer 2, MAC is divided into eMAC (expressMAC) and pMAC (preemptableMAC) logically. The eMAC processes the ST frame at high speed, and the pMAC stores the divided frames in the buffer, merges them into a complete frame, and sends it up to the upper layer. The buffer is located in the receiving MAC merge sublayer, and the fragmented frames are merged in the buffer and sent to the pMAC. If it is not a fragmented frame, it is sent directly to the pMAC without buffer storage.

**Figure 4:**
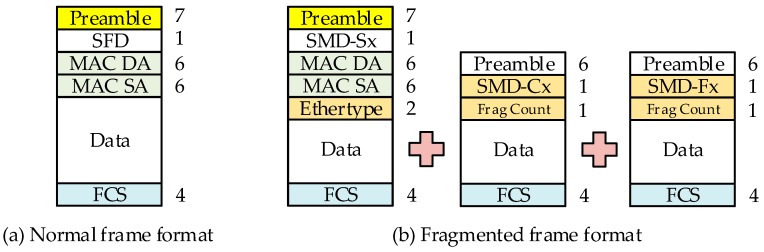
IEEE 802.1Qbu frame format.

When transmitting a frame to the pMAC, it is necessary to know whether the frame is a fragmented frame, and how many frames are fragmented. This information, together with the frame preemption, consists of additional formats provided by IEEE 802.1Qbu and IEEE802.1br (see Figure 4). The format includes the beginning, end, and the sequence information identifier of the fragmented frame (see Table 2). The frame is split according to this format, and the fragmented frame is restored to the original frame at the receiving node with reference to the identifier (see Figure 3). As the development progresses, the frame may be divided more than necessary. In order to control this problem, another parameter is configured to limit the maximum number of times that one frame can be divided.

## 4. Simulation

### 4.1. IVN Model Design

**Figure 5:**
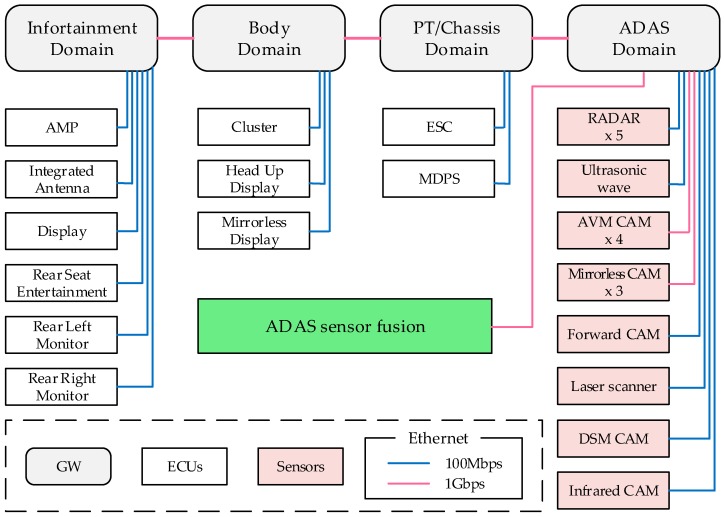
IVN model for autonomous driving vehicle.

An IVN model was designed based on the traffic required for autonomous driving vehicles (see Figure 5). The basic IVN structure and traffic information were provided by a major motor company in Korea, and we redesigned the IVN of each domain based on the traffic information. To illustrate the overall IVN structure, we configured a vehicle to four domains (Infotainment, Body, PT/Chassis, ADAS). Each domain had ECUs and sensors, and a gateway (GW) for each part of the vehicle. GWs are used to interconnect other domains as necessary. In addition, the ADAS sensor fusion was designed as an ECU for autonomous driving. The ADAS sensor fusion ECU collects the information from the sensors, analyzes the information from adjacent vehicles and the road environment, and extracts information necessary for driving. This information leads to control commands necessary for autonomous driving. The IVN model was configured so that the sensors required for autonomous driving were continuously generating data (see Table 3). The sensors included radio detection and ranging (RADARs) and light detection and ranging (LIDARs). The RADAR was used to determine the distance, direction, and altitude of nearby vehicles and the LIDAR was used to identify the surrounding routes and road conditions. The AVM (around view monitoring) is a vehicle camera information system that can see 360 degrees outside the vehicle in the driver’s seat. The DSM (driver status monitoring) is also a camera information system which was mounted inside the vehicle to recognize the pupil and prevent drowsy driving. In addition, ECUs for infotainment of vehicles and ECUs for stream information coming from outside the vehicle for control, safety, etc., were arranged.

### 4.2. Configuring Parameter Value

Prior to the overall simulation test, it was necessary to measure the change in end-to-end delay according to the preemption guard band size, one of the variable parameters. As described above, using preemption reduces E2E delay and increases network efficiency. The most significant factor in preemption is the size of the guard band that forms the time window between the ST and the non-ST. Depending on the size of the guard band, more non-STs may be sent, so that the segmented traffic is fully integrated and can reach the destination quickly. Therefore, we conducted experiments to see how the delay changed according to the setting of the guard band size when transmitting ST and AVB streams for a simulation time of 5 s. We specified two streams (Stream 1, 2 in Table 4) and measured the preemption occurrence frequency and delay variation at the point where they intersected (see Table 5). The transmission period of the ST stream was set to 10 ms, and the transmission period of the AVB stream was set to 125 μs, which conforms to the transmission requirements of the SR Class A. The minimum guard band size was set to 80 bytes (minimum 64 bytes of frame processing plus additional header information), and the maximum guard band size was set to 512 bytes.

**Figure 6:**
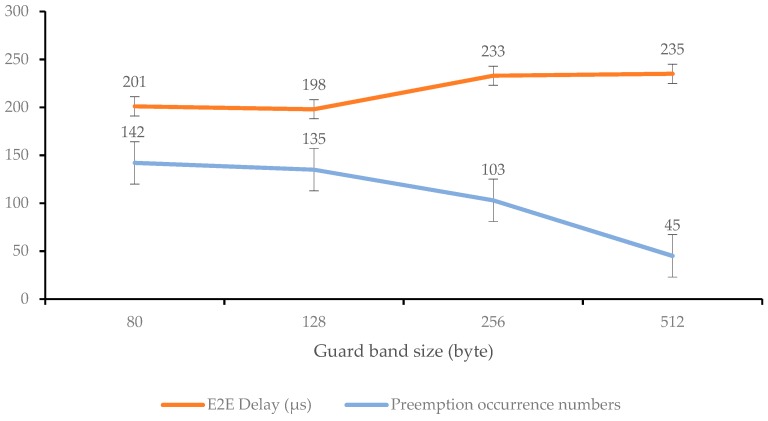
E2E delay and preemption frequency variation according to guard band size.

Experimental results show that if the guard band becomes too large or small, the frequency of preemption decreases or increases, resulting in poor data transmission efficiency. Taken together, it can be concluded that the optimal guard band size is 128 bytes (see Figure 6). Based on these experimental results, the overall TSN performance test was conducted with the guard band size of 128 bytes.

### 4.3. Performance Test on TSN Integrated Environment

For the TSN performance experiment, the stream of each ECU and sensor was allocated after the design of the IVN model. It was divided into a total of 16 streams, and periodic data transmission was performed by specifying the source to the destination according to the ST and AVB transmission formats defined in the TSN standard (see Table 3). Depending on the IVN domain, links exceeding 100 Mbps were replaced by 1 Gbps links. This is because relatively large video and voice data are mixed in the domain of ADAS and Infotainment. The size of the guard band was set to 128 bytes, which was derived as the optimal value.

Experimental results show that applying the TSN preemption in combination with ST transmission was more efficient in terms of the E2E delay than using AVB only. It was shown that the delay was reduced in most cases except for streams 5 and 12. Concerning the difference of E2E delay in Table 4, the delay was not reduced due to the fact that the preemption occurred more frequently than necessary under too much traffic by chance. Based on this result, it can be inferred that the proper spacing of the ST, placement of the IVN link, and setting of the guard band size in the preemption are important for IVN design for autonomous driving. As a result of comparing the E2E delays between an AVB and TSNs through experiment, it was confirmed that TSNs satisfy the ST transmission requirements and the frame preemption required to compensate for the delay time caused by AVB stream transmission interruptions (see Table 4).

Through this experiment, it was found that the results can vary greatly depending on how the traffic is arranged and the parameter values are configured in the environment where the functions of TSN standards are integrated. In the future, it may be necessary to modify some of the requirements or recommended parameters of existing standards. This is because the performance of TSNs may not be as desirable when various standards, such as the queuing policies and the link speed configurations of IEEE 802.3 with speeds of 10, 100 Mbps, or greater than 1 Gbps, are integrated into newly revised 802.1Q. We will proceed with further implementation and experimentation to address these issues.

## 5. Conclusions

In this paper, a simulator was developed by integrating the main TSN functions under development. Based on the ST for streaming and control data from various sensors provided by a major motor company in Korea, we constructed a TSN in a vehicle for autonomous driving. When testing various standard functions in the integrated environment, it was found that the TSN time-division transmission method was required to satisfy the requirements and that the preemption was required to compensate for the AVB delay. Also, the optimal size of the guard band was found to be 128 bytes for autonomous vehicles through analysis. We anticipate that various future standards for TSNs will be published and that when these standards are applied to an integrated environment, the requirements presented in the existing standards will need to be reconsidered.

In the future, we plan to integrate key features of the upcoming standards such as IEEE 802.1Qci, 802.1Qch, 802.3cg, 802.3ch, and so forth into the simulator developed. This next research topic would include additional performance tests by collecting vehicle traffic data from vehicle original equipment manufacturers (OEMs).

## Figures and Tables

**Table 1:** Development status of time-sensitive network (TSN) standards.

Standard	Status	Functionality
IEEE 802.1Qbv-2015	Published	Enhancements for Scheduled Traffic
IEEE 802.1Qca-2015	Published	Path Control and Reservation
IEEE 802.1Qbu-2016	Published	Frame Preemption
IEEE 802.1Qch-2017	Published	Cyclic Queuing and Forwarding
IEEE 802.1Qci-2017	Published	Per-Stream Filtering and Policing
IEEE 802.1CB-2017	Published	Frame Replication and Elimination for Reliability
IEEE 802.1CM-2018	Published	Time-Sensitive Networking for Front Haul
IEEE P802.1AS-Rev	Ongoing	Timing and Synchronization for Time-Sensitive Applications
IEEE P802.1Qcc	Ongoing	Stream Reservation Protocol (SRP) Enhancements and Performance Improvements
IEEE P802.1AX-Rev	Ongoing	Link Aggregation
IEEE P802.1Qcr	Ongoing	Asynchronous Traffic Shaping

**Table 2:** Fragmented frame identifier.

Identifiers	Functions
SMD-Sx	Field to check first fragmented frame
SMD-Cx	Field to check if the fragment is continuing
SMD-Fx	Field to check last fragmented frame
Frag Count	Fragmented frame sequence

**Table 3:** Background stream for advanced driving assistant system (ADAS) sensor fusion.

Sensor	Type	Stream Size	Number of Stream
RADAR	AVB	10 Mbps	5
Ultrasonic wave	AVB	15 kbps	1
AVM CAM	AVB	80 Mbps	4
Mirrorless CAM	AVB	80 Mbps	3
Forward CAM	AVB	80 Mbps	1
Laser scanner	AVB	10 Mbps	1
DSM CAM	AVB	80 Mbps	1
Infrared CAM	AVB	80 Mbps	1

**Table 4:** TSN streams for autonomous driving.

	Stream Info	Type	Source	Destination	Size (Byte)	Bandwidth Allocation (bps)	E2E Delay (μs)		* Diff. (μs)
							Gen1	Gen2	
1	Navigation/AVM	ST	Infotainment	ADAS	60	704 k	98.5	64.5	34
2	Navigation data	AVB	Infotainment	HUD	2	80 M	334.5	198	136.5
3	Navigation/Audio	AVB	Infotainment	Cluster	2	48 k	217.8	110	107.8
4	Audio	AVB	Infotainment	AMP	11	125 k	200	98.5	101.5
5	Entertainment	AVB	Infotainment	RSE	1250	125 k	120	120.9	−0.9
6	Entertainment	AVB	RSE	Rear Left Monitor	1250	80 M	212.9	212	0.9
	Entertainment	AVB	RSE	Rear Right Monitor	1250	80 M	212.9	212	0.9
7	GPS/V2X	AVB	Integrated Antenna	Infotainment	16	1 M	54.4	54.4	0
8	Control data	ST	Body	PT/Chassis	4	3 k	101	50.9	50.1
9	PT/Chassis info	ST	PT/Chassis	ADAS Sensor fusion	230	184 k	100	50.2	49.8
10	ADAS sensor fusion	AVB	ADAS Sensor fusion	Cluster	2	125 k	125	123.3	1.7
11	ADAS sensor fusion	AVB	ADAS Sensor fusion	HUD	2	125 k	125	123.3	1.7
12	Forward camera	AVB	ADAS Sensor fusion	Integrated Antenna	250	80 M	315.4	345.6	−30.2
13	Control data	ST	ADAS Sensor fusion	ESC	625	500 k	99.3	57.1	42.2
14	Control data	ST	ADAS Sensor fusion	MDPS	625	500 k	99.3	57.1	42.2
15	AVM image	AVB	ADAS Sensor fusion	Display	1250	80 M	210.7	209.8	0.9
16	Mirrorless image	AVB	ADAS Sensor fusion	Mirrorless Display	1250	80 M	205.7	199	6.7

* Diff. = Difference of E2E delay.

**Table 5:** E2E delay variation according to guard band size.

Preemption		X	O			
Guard band size (byte)		-	80	128	256	512
E2E Delay (μs)	ST	64.5	64.5	64.5	64.5	64.5
	AVB	234	201	198	233	235
